# Association of Dietary Inflammatory Index with cardiovascular disease in Kurdish adults: results of a prospective study on Ravansar non-communicable diseases

**DOI:** 10.1186/s12872-020-01707-7

**Published:** 2020-10-07

**Authors:** Azad Ayeneh pour, Mehdi Moradinazar, Mehnoosh Samadi, Behrooz Hamzeh, Farid Najafi, Sheno Karimi, Fakhereh Faraji, Mitra Darbandi, Yahya Pasdar

**Affiliations:** 1 Departmalet of Nutrition, School of Nutritional Science and Food Technology, Kermanshah University of Medical Sciences, Kermanshah, Iran; 2 Research Center for Environmaletal Determinants of Health (RCEDH), Health Institute, Kermanshah University of Medical Sciences, Kermanshah, Iran; 3 Social Developmalet and Health Promotion Research Center, Health Institute, Kermanshah University of Medical Sciences, Kermanshah, Iran; 4 Cardiovascular Research Center, Health Institute, Kermanshah University of Medical Sciences, Kermanshah, Iran; 5 Clinical Research Developmalet Center, Taleghani and Emam Ali Hospital, Kermanshah University Of Medical Sciences, Kermanshah, Iran

**Keywords:** Dietary inflammatory index, Cardiovascular diseases, Diet, Calorie intake

## Abstract

**Background:**

Various diets and dietary compounds, through their inflammatory properties, are involved in the pathogenesis of chronic diseases including Cardiovascular Diseases (CVDs). Dietary Inflammatory Index (DII) can evaluate the inflammatory properties of diet. The purpose of this study was to determine the association between DII and CVDs in participants of the Ravansar Non-Communicable Diseases (RaNCD) cohort study, Kermanshah, Iran.

**Materials:**

The present cross-sectional study was conducted using the recruitment phase data of the RaNCD cohort study on 6369 participants aged 35 to 65 years. The Food Frequency Questionnaire (FFQ) was used to assess diet. The DII scores were calculated using FFQ data. Participants with a history of myocardial infarction, stroke and coronary artery disease, and/or taking medications for the CVDs were considered as the CVDs patients.

**Results:**

Of the 6369 studied participants, 9% (*n* = 579) had CVDs history. The mean DII score in this study was − 0.84 ± 1.6. Odds ratio (OR) of CVDs in women was 1.6 times higher than in men (CI 95% = 1.3–1.9), which this association was remained after adjusting for confounding variables (OR = 1.5, CI% = 1.2–1.9). The risk of CVDs in the fourth quartile of DII was 1.4 times higher than the first quartile of DII (OR: 1.4, CI 95% = 1.1–1.8). We found that higher adhere to DII was associated with risk of CVDs.

**Conclusion:**

According to current documents, given the role of diet through inflammatory properties on the risk of CVDs, it is recommended to use DII as an appropriate index to measure the effect of diet on CVDs in Iranian population. In addition, a diet with lower DII may be healthier diet for cardiovascular health.

## Background

Cardiovascular diseases (CVDs) are the leading cause of mortality in the worldwide [1, 2], the most important cause of disability, and a decline in quality of life [3]. One of the risk factors for CVDs is dietary pattern or diet. Since the diet is both effective in chronic diseases [2] and can be modulatory to inflammatory conditions [4, 5], it can therefore act as a mediator between chronic diseases including CVDs and chronic inflammation. Traditional diets, such as Mediterranean diets rich in fruits and vegetables, whole grains, olive oil and low amounts of refined grains and processed foods, are good sources of inflammation-modulating nutrients [5, 6]. Diet has been associated with reduced CVDs risk as well as lower C-reactive protein (CRP) levels [4]. In contrast, Western diets that are rich in refined grains, simple carbohydrates, fats, and high-fat dairy are associated with increased levels of inflammatory markers including CRP and Interleukin-6 (IL-6) [4, 7].

Since nutrients are not consumed on their own and overlap with their potential benefits or disadvantages, analyzing dietary patterns and assessing the overall inflammatory potential of the diet are useful approaches to assess the association between diet and chronic disease risk [8, 9].

Dietary Inflammatory Index (DII) is an indicator for assessing the inflammatory potential of a person’s diet [10]. The validity of DII has been investigated with two dietary evaluation methods, which have shown that the DII is capable of predicting hs-CRP -This test accurately measures the low level of C-reactive protein. The hs-CRP can be used to detect the risk of cardiovascular disease- concentration with a cut-off point of values less than or equal 1 and greater than 3 mg/l. A high score of DII (pro-inflammatory diet) is associated with hs-CRP higher than 3 mg/l [11]. In recent years, several studies have been conducted on the association between chronic inflammation, diet, and CVDs [2, 12], but since different diet and dietary habits exist in different societies, and especially in various ethnicities and races, specific studies in ethnic subgroups can accordingly provide appropriate evidence regarding the planning and design of preventive interventions in relation to the CVDs. This study is one of the first studies on a significant population of Kurdish people in Iran. The purpose of this study was to determine the association between DII and CVDs in participants of the Ravansar Non-Communicable Diseases (RaNCD) cohort study, Kermanshah, Iran.

## Methods

### Study design

This is a cross-sectional study. We use the baseline data of Ravansar Non-Communicable Disease (RaNCD) cohort study to conduct this study. RaNCD cohort is a part of Prospective Epidemiological Research Studies in IRAN (PERSIAN) conducted on various ethnicities of Iranian population in coordination with Ministry of Health and Medical Education in which 10,000 adults were recruited for RaNCD. Ravansar is one of the cities of Kermanshah province. 30% of Ravansar population is the 35–65 years old both in rural and urban areas, mainly from Iranian Kurdish ethnicity. Further details of the RaNCD study have already been published [13, 14].

### Inclusion and exclusion criteria

Eligibility criteria in the cohort study comprised being in the age range of 35–65 years, permanent inhabiting the Ravansar region (Ravansar town and all villages in its vicinity), having Iranian nationality. For the purpose of this study, participants with renal failure and kidney stones (*n* = 1357), cancer (*n* = 93) liver disease (*n* = 817), thyroid disease (*n* = 727) and inflammatory diseases (*n* = 310), pregnant women (*n* = 88) and those with missed information (*n* = 304) were not included to this study. Finally, out of 10,065 participants in RaNCD cohort study, 6369 persons (3223 men and 3146 women) were included.

### Data collection and measurement

Data collection and all measurements were conducted and assessed in the RaNCD cohort site. Invitation method had done through a face-to-face appointment via visiting the potential participant at home [14].

The wealth index was measured, using 15 items (including housing, car, washing machine, dishwasher, Freezer, computer, internet access, motorcycle, car rental, car type, vacuum cleaner,color TV, TV type, bathroom, cell phone) by principal component analysis (PCA) method then wealth index was categorized into five groups, from the poorest to the richest [15].

The non-smoker was defined people who reported they had not smoked. Current smokers were people who reported they had smoked at least 100 cigarettes and former smokers were those who had quit with a history of smoking at least 100 cigarettes during their lifetime [16].

The Physical Activity Questionnaire is a standardized cohort study questionnaire based on met/hour per day [17].

The standardized 137-item 1-year food frequency questionnaire (FFQ) [14] of PERSIAN cohort study was completed to evaluate the diet. Frequency of consumption and size of common share were considered for each food item. Updated dietary databases were used to calculate the amount of nutrient intake [14, 18]. The FFQ was used to calculate DII. The method for calculation of DII has been described in various reports [2, 19]. The DII was suggested by reviewing articles published between 1950 and 2010 on the association between a variety of dietary parameters and 6 inflammatory markers (IL-1β, IL-4, IL-6, IL-10, CRP and TNF-α). Accordingly, 45 dietary parameters, including macronutrients, micronutrients, flavonoids and other food items, have been identified that can have inflammatory effects. The inflammatory potential of each parameter was assessed by their effect on increasing, decreasing or not affecting the levels of these inflammatory markers. If each of these food items increased the levels of inflammatory markers, they would score + 1, if they decreased the levels of inflammatory markers, they would score − 1, and if they had no effect on the levels of inflammatory markers, they would receive an inflammatory score of 0. The DII score can range between − 8.87 (the highest anti-inflammatory score) and + 7.98 (the highest pro-inflammatory score). On the basis of mean intake and global standard deviation, Zscore was determined for each parameter. Then, the Z-score became a percentile. The inflammatory score for each of the dietary parameters was calculated by this manner, and then the inflammatory score of all parameters was summed to calculate the total DII score. The more negative the DII score, the more powerful anti-inflammatory property and the more positive values, the more powerful pro-inflammatory characteristics [8, 20].

According to the RaNCD cohort study protocol, the CVDs participants are people with a history of hospitalization and/or treatment for one or more heart diseases such as stroke, MI and coronary artery disease, and/or taking medications for the CVDs.

Diagnosis of type II diabetes includes fasting blood sugar (FBS) levels equal to or greater than 126 mg/dl and/or treatment with hypoglycemic drugs. Also, subjects with systolic blood pressure equal to or greater than 140 mmHg and diastolic blood pressure equal to or greater than 90 mmHg, and/or those treated with blood pressure lowering medications were considered as subjects with HTN.

In this study, dyslipidemia was also considered to be a disorder of serum lipid profile indices including one or more of the following: LDL > 130 mg/dl, HDL < 45 mg/dl, TG > 150 mg/dl, Total Cholesterol > 200 mg/dl and/or taking blood lipid lowering medications including amlodipine, atorvastatin, clofibrate, fenofibrate, gemfibrozil, lovastatin and simvastatin [14].

### Data analysis

Data were described using mean and standard deviation for quantitative variables and frequency and percentage for qualitative variable. The crude ORs with 95% confidence intervals used to examine the relationship between DII on prevalence of CVDs. Variables with *p* < 0.3 in univariable analysis were entered into multivariable logistic model. Then, variables with *p*-value> 0.05 were removed using forward or backward method. The fractional polynomial method was performed to quantitatively associate the effect of DII on prevalence of CVDs. In this method, the effects of demographic variables and BMI on CVDs were first adjusted. The effect of DII was then evaluated. The fractional polynomial is a regular polynomial alternative method that provides flexible parameterization for continuous variables. In all of the analyses, missing values was deleted (less than 1%). All analyzes were performed using Stata version 14.1 software (Stata Corp, College Station, TX, USA) with 95% confidence interval.

## Results

Of the 6369 studied, 3223 (50.6%) were men. The mean age of participants was 46.9 ± 8.4 years and 46.21% of participants were in the age group of 35–45 years. Almost 90% of participants were married and the majority (56.63%) was urban. The mean energy 1 intake in the participants was 3010 ± 1039.9 kcal/day, 58.65% of which was carbohydrate and this difference was statistically significant in different quartiles of DII (*P* < 0.001). The mean energy intake in the first quartile was significantly higher than the fourth quartile of DII (P < 0.001). The mean carbohydrate energy in the fourth quartile was significantly higher than the first quartile of DII. However, the mean energy of protein and fat in the first quartile of DII was significantly higher than the fourth quartile. Of the total population, 26.1% of participants had low physical activity, which was significantly different in the different quartiles of DII (*P* < 0.001). Most people with high physical activity were in the Q1 (the most powerful anti-inflammatory diet).

The prevalence of CVD in the present study was 9.1% and its prevalence in first to fourth quarters were 22.45, 21.42, 23.49 and 32.64%, respectively (*P* < 0.05).

Moreover, 35.8% of women were in the highest quartile of DII (the most powerful pro13 inflammatory diet), while only 14.15% of men were in this quartile. People with higher educational level had a lower DII than those with lower educational level. People with the highest socioeconomic status had a significantly lower DII (*P* < 0.001) than those with the lowest level. Additionally, 13.94% of the studied subjects had HTN and these individuals had significantly higher DII than those who had no HTN (P < 0.001). Furthermore, 43.38% of the subjects had dyslipidemia, most of whom (26.9%) were in the fourth quartile of DII. Approximately 7.44% of the subjects had diabetes; there was no significant difference in the DII score between the two groups with and without diabetes (*P* = 0.795) (Table 1).

**Table 1:** Demographic characteristics of participants based on Dietary Inflammatory Index in RaNCD cohort study

Variable		Total	Q1*	Q2	Q3	Q4**	*P* value
N (%)		6369 (100)	1554 (24.40)	1663 (26.11)	1569 (24.63)	1583 (24.85)	
Mean (min,max)		−0.84(−4.5,+ 4.6)	−2.8(− 4.5,-2.0)	−1.5(− 2.0,-1.0)	−0.44(− 1.0,+ 0.25)	1.4(+ 0.25,+ 4.6)	
Gender	Men	3223 (50.60)	1033 (32.06)	1005 (31.18)	729 (22.61)	456 (14.15)	< 0.001
	Women	3146 (49.40)	521 (16.56)	658 (20.91)	840 (26.70)	1127 (35.83)	
Age	35–45	2943 (46.21)	775 (26.33)	854 (29.02)	724 (24.60)	590 (20.05)	< 0.001
	46–55	2013 (31.61)	496 (24.63)	500 (24.84)	503 (24.99)	514 (25.54)	
	56–65	1413 (22.19)	283 (20.02)	309 (21.87)	342 (24.21)	479 (33.90)	
Marital status	Married	5696 (89.43)	1462 (25.67)	1526 (26.80)	1399 (24.56)	1309 (22.97)	< 0.001
	Single	327 (5.13)	42 (12.84)	69 (21.10)	85 (26.00)	131 (40.06)	
	Divorced	346 (5.44)	50 (14.45)	69 (19.84)	85 (24.46)	143 (41.25)	
Education years	Illiterate	1576 (24.74)	252 (16.00)	309 (19.60)	375 (23.80)	640 (40.60)	< 0.001
	1–5	2366 (37.15)	505 (21.34)	598 (25.28)	629 (26.58)	634 (26.80)	
	6–9	1094 (17.18)	329 (30.08)	326 (29.79)	269 (24.58)	170 (15.55)	
	10–12	840 (13.19)	284 (33.80)	262 (31.20)	200 (23.81)	94 (11.19)	
	≥13	493 (7.74)	184 (37.32)	168 (34.08)	96 (19.48)	45 (9.12)	
SES	Poor	1346 (21.13)	209 (15.53)	279 (20.72)	348 (25.85)	510 (37.90)	< 0.001
	2	1299 (20.40)	261 (20.10)	300 (23.10)	337 (25.94)	401 (30.86)	
	3	1287 (20.21)	272 (21.14)	352 (27.35)	349 (27.11)	314 (24.40)	
	4	1239 (19.45)	361 (29.13)	373 (30.10)	285 (23.00)	220 (17.75)	
	Rich	1198 (18.81)	451 (37.65)	359 (29.97)	250 (20.87)	138 (11.51)	
Place	City	3607 (56.63)	1197 (33.18)	1064 (29.50)	802 (22.23)	544 (15.09)	< 0.001
	Village	2762 (43.37)	357 (12.92)	599 (21.68)	767 (27.77)	1039 (37.63)	
Physical Activity	1	1662 (26.10)	427 (25.70)	458 (27.55)	389 (23.40)	388 (23.35)	< 0.001
	2	3255 (51.11)	725 (22.28)	794 (24.40)	829 (25.46)	907 (27.86)	
	3	1452 (22.80)	402 (27.68)	411 (28.30)	351 (24.18)	288 (19.84)	
Smoking	No	5037 (79.09)	1176 (23.35)	1275 (25.31)	1261 (25.04)	1325 (26.30)	< 0.001
	Current	807 (12.67)	222 (27.50)	240 (29.74)	197 (24.43)	148 (18.33)	
	Former	525 (8.24)	156 (29.70)	148 (28.20)	111 (21.15)	110 (20.95)	
BMI	< 18.49	128 (2.01)	16 (12.5)	26 (20.32)	39 (30.47)	47 (36.71)	< 0.001
	18.5–24.9	1939 (30.44)	428 (22.08)	478 (24.65)	480 (24.75)	553 (28.52)	
	25–29.9	2734 (42.93)	677 (24.76)	770 (28.16)	654 (23.93)	633 (23.15)	
	30–34.9	1257 (19.74)	355 (28.24)	309 (24.58)	306 (24.35)	287 (22.83)	
	≥35	311 (4.88)	78 (25.08)	80 (25.73)	90 (28.94)	63 (20.25)	
Hypertension	No	5481 (86.06)	1361 (24.84)	1451 (26.47)	1343 (24.50)	1326 (24.19)	0.007
	Yes	888 (13.94)	193 (22.72)	212 (23.87)	226 (24.46)	257 (28.95)	
Dyslipidemia	No	3606 (56.62)	819 (22.71)	910 (25.24)	907 (25.15)	970 (26.90)	0.000
	Yes	2763 (43.38)	735 (26.60)	753 (27.25)	662 (23.95)	613 (22.20)	
Diabetes	No	5895 (92.56)	1436 (24.35)	1542 (26.15)	1445 (24.52)	1472 (24.98)	0.795
	Yes	474 (7.44)	118 (24.90)	121 (25.52)	124 (26.17)	111 (23.41)	
CVD	No	5790 (90.9)	1424 (24.59)	1539 (26.58)	1433 (24.75)	1394 (24.08)	< 0.001
	Yes	579 (9.1)	130 (22.45)	124 (21.42)	136 (23.49)	189 (32.64)	
Calorie intake	Mean (SD)	3010.3 (1039.9)	3914.35 (950.1)	3357.6 (808.7)	2791.8 (673.2)	1974.7 (529.8)	< 0.001
CHO Kcal	Mean (SD)	58.65 (6.08)	57.69 (5.53)	58.60 (5.65)	58.75 (5.90)	59.56 (6.99)	< 0.001
Pro Kcal	Mean (SD)	13.3 (2.3)	13.7 (2.2)	13.5 (2.1)	13.3 (2.3)	12.7 (2.5)	< 0.001
Fat Kcal	Mean (SD)	28.0 (5.49)	28.5 (4.8)	27.8 (5.0)	27.9 (5.4)	27.7 (6.4)	< 0.001

The mean DII score in this study was − 0.84 ± 1.6 and the DII score ranged from − 4.5 (the most powerful anti-inflammatory diet) to + 4.6 (the most powerful pro-inflammatory diet).

### DII and CVD risk

The odds ratio of CVDs was 1.6 times higher in women than in men (CI = 1.3–1.9) and remained significant after adjusting for confounding variables (OR = 1.5, CI = 1.2–1.9). The odds ratio of CVDs increased with age, so that it was 3.2 times in the age group of 46–55 years (CI = 2.4–4.1) and 8.6 times in the age group of 56–65 years (CI = 6.7–11.0) higher than in the age group of 35–45 years, and this association was significant after adjusting the confounding variables. Those who were physically more active had a lower odd for CVDs. The risk of CVDs was increased with increasing body weight and BMI, so that those with BMI ≥ 35 had an odds of 6.8 times higher than those with BMI < 18.9 (CI = 2.0–22.5). The energy intake and percentage of energy intake from carbohydrates both with OR = 0.99 prevented the CVDs, whereas percentage of energy intake from protein (OR = 1.02, CI = 0.98–1.05) and fat (OR = 1, CI = 0.99–1.02) were risk factors for the CVDs.

People with HTN had significantly higher odds ratio of CVDs (OR = 40.5, CI = 32.6–50.3) than those without HTN; this association was remained after control of confounding variables (OR = 28.6, CI = 22.7–36).

People with dyslipidemia had a 1.9-fold higher risk of CVDs; the odds 16 ratio remained significant after adjusting the confounding variables (OR = 1.6, CI = 1.3–2.0). In the univariate analysis, the risk of CVDs was 3.8 times higher in individuals with diabetes than in those without diabetes (CI = 3.0–4.8), but no significant association was found after adjusting the confounding variables (Table 2).

**Table 2:** Odds ratios for cardiovascular diseases in RaNCD cohort study

Variable		Total N(%)	Crud OR(95% CI)	Adjusted OR(95% CI)
Gender	Men	227/3223(%7.0)	1	1
	Women	352/3146(%13.9)	1.6 (1.3–1.9)	1.5 (1.2–1.9)
Age	35–45	90/2943(%3.0)	1	1
	46–55	186/2013(%11.0)	3.2 (2.4–4.1)	1.2 (0.9–1.5)
	56–65	303/1413(%24.0)	8.6 (6.7–11.0)	1.6 (1.3–2.1)
Marital status	Married	516/5696(%11.0)	1	1
	Single	2/327(%1.4)	0.06 (0.01–0.24)	0.3 (0.1–0.8)
	Divorced	61/346(%19.0)	2.1 (1.6–2.8)	1.0 (0.7–1.4)
Education years	Illiterate	260/1576(%19.0)	1	1
	1–5	196/2366(%10.3)	0.4 (0.3–0.5)	1.09 (0.87–1.35)
	6–9	59/1094(%7.2)	0.28 (0.21–0.38)	1.20 (0.87–1.66)
	10–12	41/840(%5.9)	0.25 (0.18–0.36)	1.26 (0.84–1.89)
	≥13	23/493(%5.6)	0.24 (0.15–0.38)	0.99 (0.59–1.65)
SES	Poor	150/1346(%12.8)	1	1
	2	138/1299(%12.8)	0.9 (0.7–1.2)	1.12 (0.87–1.44)
	3	97/1287(%10.5)	0.6 (0.4–0.8)	1.06 (0.82–1.38)
	4	111/1239(%10.8)	0.7 (0.6–1.0)	1.25 (0.96–1.64)
	Rich	83/1198(%8.5)	0.5 (0.4–0.7)	1.34 (0.99–1.81)
Place	City	316/3607(%10.3)	1	1
	Village	263/2762(%12.1)	1.09 (0.9–1.3)	1.18 (0.99–1.40)
Physical Activity	1	195/1662(%13.8)	1	1
	2	298/3255(%11.3)	0.7 (0.6–0.9)	0.7 (0.5–0.9)
	3	86/1452(%7.0)	0.4 (0.3–0.6)	0.6 (0.4–0.8)
Smoking	No	450/5037(%11.0)	1	1
	Current	49/807(%7.7)	0.6 (0.4–0.8)	0.88 (0.65–1.2)
	Former	80/525(%16.1)	1.8 (1.4–2.3)	1.13 (0.86–1.49)
BMI	< 18.49	3/128(%4.1)	1	1
	18.5–24.9	122/1939(%7.5)	2.7 (0.8–8.9)	1.3 (0.55–3.28)
	25–29.9	250/2734(%10.9)	4.1 (1.3–13.2)	1.4 (0.58–3.42)
	30–34.9	160/1257(%14.9)	6.0 (1.9–19.3)	1.4 (0.57–3.45)
	≥35	44/311(%16.0)	6.8 (2.0–22.5)	1.38 (0.54–3.50)
Hypertension	No	133/5481(%3.4)	1	1
	Yes	446/888(%52.5)	40.5 (32.6–50.3)	28.6 (22.7–36.0)
Dyslipidemia	No	243/3606(%8.0)	1	1
	Yes	336/2763(%84.0)	1.9 (1.6–2.2)	1.6 (1.3–2.0)
Diabetes	No	462/5895(%7.0)	1	1
	Yes	117/474(%28.9)	3.8 (3.0–4.8)	1.8 (1.3–2.4)
DII	Q1	130/1554(%9.3)	1	1
	Q2	124/1663(%9.2)	0.8 (0.6–1.1)	0.7 (0.5–1.0)
	Q3	136/1569(%11.0)	1.0 (0.8–1.3)	0.6 (0.4–0.9)
	Q4	189/1583 (%15.2)	1.4 (1.1–1.8)	0.8 (0.5–1.2)
Calorie intake	Increase one unit	–	0.99 (0.9995–0.9997)	0.9999 (0.9998–1.00)
CHO Kcal	Increase one gram	–	0.99 (0.97–1.00)	0.99 (0.98–1.01)
Pro Kcal	Increase one gram	–	1.02 (0.(0.98–1.05)	0.99 (0.95–1.02)
Fat Kcal	Increase one gram	–	1.00 (0.98–1.02)	1.00 (0.99–1.02)

Overall, a direct association was observed between DII and CVDs, with odds ratio of CVDs in the fourth quartile being 1.4 times higher than in the first quartile of DII (CI = 1.1–1.8), but this association was not significant after adjusting the confounding variables (Table 2).

**Fig. 1:**
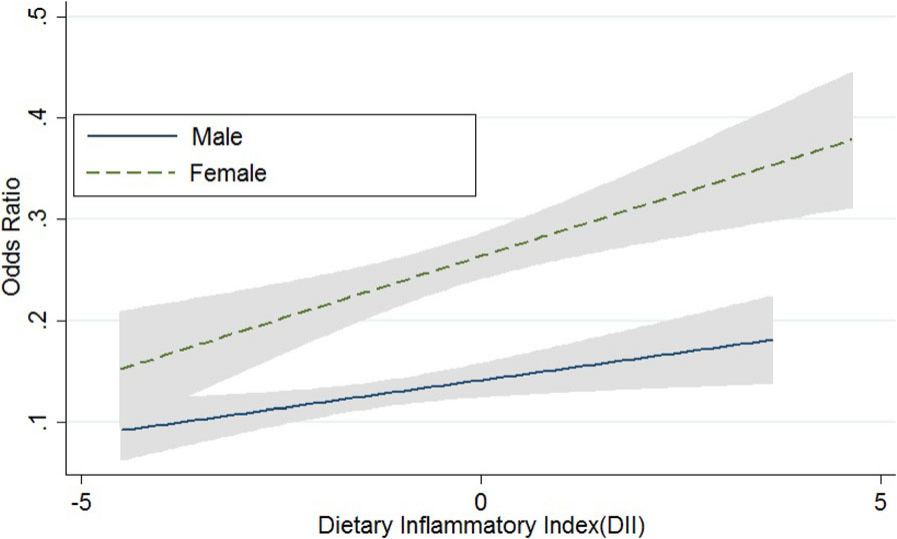
The Association of Cardiovascular Disease with Dietary Inflammatory Index

Figure 1 shows the dose-response relationship between DII and odds ratio of CVDs. After controlling the effects of demographic variables and BMI, with increasing DII, the odds ratio of CVDs was increased in both sex groups. In addition to the greater magnitude of this increase in women than in men, the effect of DII on women with heart disease was also greater.

## Discussion

The results of our study showed that the prevalence of HTN and dyslipidemia was higher in the upper quartiles of DII than in the lower quartiles. In a cross-sectional study in Luxembourg, the prevalence of dyslipidemia in high quartiles of DII was also higher than in low quartiles [21]. In a study in France, there was also a significant association between increased DII score and the risk of metabolic syndrome, HTN, and increased triglyceride, but no significant association was reported with FBS and waist circumference [12]. In the present study, the obesity had a direct and significant association with CVDs and DII, so that people with the most powerful pro inflammatory diet were obese and at higher risk of CVDs. Ruiz-Canela et al. also observed an association between higher DII score and obesity, especially abdominal obesity [8]. As BMI and prevalence of overweight and obesity have increased with increasing DII quartiles, it can be expected that the prevalence of dyslipidemia and HTN is also higher in the fourth quartile than in the first quartile. Given that the level of physical activity decreased with increasing DII quartiles one might expect a higher prevalence of obesity and dyslipidemia in the fourth quartile than in the first quartile.

In the present study, the mean energy intake in the fourth quartile was significantly lower than the first quartile of DII. Bawaked et al. support the results of ours. Both in the adult population and in children, a characteristic low-energy diet is usually a diet rich in healthy foods, including fruits and vegetables. In the present study, similar to the study of Bawaked et al. the inflammatory potential of the diet was increased with increasing energy density, but the energy intake was decreased. It should be noted that a high energy density diet does not necessarily have high energy intake compared to a low energy density diet because there are a large number of nutrients with different total energy content per unit of energy density [22].

The prevalence of CVDs risk factor, including obesity (70%), HTN (61%) and dyslipidemia (62%) was high in women. In this study, the risk of CVDs in women was 1.6 times higher than in men, which may be due to the higher prevalence of CVDs risk factors. One reason for the higher reported CVDs in women than in men may be that they are more likely to visit a doctor and take medication because women are more likely to seek medical attention than men and are more likely to visit a physician. In fact, in RaNCD cohort study the definition of CVDs is partly based on self-reported data in addition to CVDs-drug specific consumption. It is worth noting that inappropriate one dietary pattern in women can also be important causes of their higher prevalence of CVDs than in men.

In the present study, the level of inflammatory markers has not been measured but since obesity is actually a type of inflammatory condition in the body and as the prevalence of obesity has increased with increasing quartiles of DII, it can be concluded that the results of this study show association of DII with inflammatory conditions. Similar to the results of the present study, Fung et al. observed the association of dietary indicators, including the Alternate Healthy Eating Index (AHEI) and the alternative Mediterranean Diet Score (aMED) with inflammatory markers including CRP [23]. Pimenta et al. and Alkerwi Aa and et al. found no significant association between DII and metabolic syndrome components [21, 24]. In these studies, no significant association was also found between DII and metabolic syndrome components [24]. However, the results of some studies contradict the results of this research. For example, in a study of Wirth et al. in the USA, there was a significant association between abnormal blood glucose levels with increased DII [7]. One of the reasons could be the in vitro differences in measuring the blood glucose levels in people with diabetes, as well as differences in studies defining diabetes or differences in the criteria that determine diabetes, which have led to different results.

One of the limitations of the present study was the impossibility of investigating the association between DII and serum levels of inflammatory markers, as serum levels of these markers were not measured in the early phase of the RaNCD cohort study. Another limitation of present study was the effect of genetic and biological differences on the results, that a homogeneous population with a high sample size was used to control these confounding factors. One of the advantages of the present study is that this is the only study aimed to determine the association of DII with CVDs among the Kurdish population in Iran with a large sample size. Other strengths of this study could be the high quality of data collection, population-based study, and adjustment of all known confounding factors including age, gender, physical activity, Wealth, BMI, energy intake, and percentage of energy derived from macronutrients. On the other hand, the use of DII rather than inflammatory markers to assess the impact of inflammation on clinical outcomes can help directly measurement of the diet impact on clinical outcomes through inflammation, and secondly reduction of the study costs. The calculation of DII through a cost-effective and non-invasive method (FFQ) can evaluate the inflammatory properties of the diet.

## Conclusion

According to current documents, given the role of diet through inflammatory properties on the risk of CVDs, it is recommended to use DII as an appropriate index to measure the effect of diet on CVDs in Iranian population. In addition, a diet with lower DII may be healthier diet for cardiovascular health.

## Data Availability

The data analyzed in the study are available from the corresponding author on reasonable request.

## Abbreviations

DII: Dietary inflammatory index
CVDs: Cardiovascular diseases
BMI: Body mass index
HTN: Hypertension
MI: Myocardial infraction
CRP: C-reactive protein
IL: Interleukin
hs-CRP: High sensitivity CRP
RaNCD: Ravansar non-communicable diseases
PERSIAN: Prospective epidemiologic research of IRAN
FFQ: Food frequency questionnaire
TNF-α: Tomor necrosis factor-α
FBS: Fasting blood sugar
LDL: Low density lipoprotein
HDL: High density lipoprotein
TG: Triglyceride
AHEI: Alternative healthy eating index
a MED: Alternative Mediterranean diet score
